# Correlation between vaginal flora and cervical immune function of human papilloma virus-infected patients with cervical cancer

**DOI:** 10.4314/ahs.v23i2.19

**Published:** 2023-06

**Authors:** Qiang Wang, Wenmin Qin, Wei Gao, Kai Zhao, Xiaohong Pan, Xiaofei Jiang, Juan Zhao

**Affiliations:** 1 Department of Gynecology and Obstetrics, Suqian Maternity Hospital, Suqian 223800, Jiangsu Province, China; 2 Department of Gynecology, Xuzhou Hospital of Traditional Chinese Medicine Affiliated to Nanjing University of Chinese Medicine, Xuzhou 221003, Jiangsu Province, China

**Keywords:** Cervical cancer, human papilloma virus, immune function, vaginal flora

## Abstract

**Background:**

To analyse the correlation between vaginal flora and cervical immune function of HPV-infected patients with cervical cancer.

**Methods:**

Six hundred females with genital tract infections treated in Xuzhou Hospital of Traditional Chinese Medicine from January 2014 to December 2016 were selected and divided into a high-risk HPV group (n=246) and a control group (n=354). The vaginal flora and human T lymphocyte subsets (CD3+, CD4+, CD8+) were detected. Multivariate logistic regression analysis was performed to explore the risk factors for HPV infection.

**Results:**

The numbers of CD4+ and CD4+/CD8+ T cells of the high-risk HPV group were significantly lower than those of the control group (P<0.05). The two groups had similar numbers of CD3+ and CD8+ T cells. In the high-risk HPV group, the positive rates of *Lactobacillus, Chlamydia trachomatis, Mycoplasma hominis, mycetes, Ureaplasma urealyticum* and bacterial vaginosis were significantly higher than those of the control group (P<0.05). There was no significant difference in the positive rates of trichomonads between the two groups. Multivariate logistic regression analysis revealed that *C. trachomatis* and *U. urealyticum* were independent risk factors for high-risk HPV infection (P<0.05).

**Conclusion:**

High-risk HPV infection in patients with cervical cancer was associated with vaginal flora and immune function. *C. trachomatis* and U. urealyticum were independent risk factors for high-risk HPV infection.

## Introduction

Human papilloma virus (HPV) is a common DNA virus in human mucosal squamous epithelium and skin. Clinical diseases, such as oropharyngeal cancer^1^, esophageal cancer, cervical cancer, genital warts, vulvar cancer, penile cancer and perianal cancer, are often associated with HPV infection^2^,^3^. Cervical HPV infection is mainly sexually transmitted, and the rate is high in sexually active females. However, HPV infection does not necessarily develop into cervical cancer, because most can clear HPV virus through their own immunity. In only a few cases, body immunity cannot remove the virus after infection, and persistent HPV infection poses a high risk for cervical cancer^4^. The female vagina is a micro-ecological system composed of endocrine regulation function, immune function, micro-ecological flora and vaginal anatomical structure. The system of normal healthy vagina is in a state of dynamic balance. The cervix, located in the vagina, is influenced by the vaginal microenvironment. When the vagina is infected or injured by external pathogens, it works through the immunity function. When the vaginal micro-ecological balance is destroyed, the infection probability of HPV increases^5^. Persistent high-risk HPV infection can accelerate destruction of the original vaginal homeostasis^6^. Carcinogenic factors, such as sexual disorder and smoking, may further dysregulate the cell cycle, inhibit apoptosis, and damage the immune defence function, promoting the onset of cervical cancer. This study analysed the vaginal flora and cervical local immune function in patients with HPV infection, aiming to provide clinical evidence for selecting appropriate treatment methods.

## Materials and Methods

### Baseline clinical data

This study was approved by the ethics committee of Xuzhou Hospital of Traditional Chinese Medicine (approval No. XHTCM201401003), and written consent was obtained from all patients. Six hundred females with genital tract infections, who were treated in our hospital from January 2014 to December 2016, were selected.

Inclusion criteria: Women with history of sexual life, and without history of using antibiotics, estrogen or progesterone within the past 2 weeks; women without sexual life, vaginal medication or washing within 3 days before sampling; sampling was performed in the non-menstrual period. Exclusion criteria: Pregnant women; women with immune, endocrine or other systemic diseases; women with vaginal prolapse. Diagnostic criteria for genital tract infections: Increased vaginal discharge, with altered characters, color and odor; pruritus vulvae; lower abdominal pain; frequent urination; urodynia.

First, 608 patients were included. After exclusion of 2 cases in pregnancy, 3 with immune diseases, 1 with endocrine disease and 2 with vaginal prolapse, 600 patients were finally included in this study.

### Collection of vaginal secretion

The vagina was exposed with a bivalve speculum to take secretion at the 1/3 segment of the vaginal wall using three sterile long cotton swabs. The first cotton swab was dipped into a sterile tube containing normal saline to observe trichomonads through direct dripping. The second cotton swab was added 10% KOH to test whether there was ammonia smell. The third cotton swab was smeared on a glass slide for gram's staining for observation of *Lactobacillus*, clue cells and mycetes under an oil immersion lens.

### Collection of cervical secretion and cervical exfoliated cells

The cervix was exposed with a bivalve speculum, and the surface mucus was wiped off using a sterile cotton ball. Then a sterile cotton swab was placed into the cervical canal, rotated a cycle, taken out 5 seconds later, and then placed into an EP tube containing normal saline to detect *Ureaplasma urealyticum, Mycoplasma hominis* and *Chlamydia trachomatis*. Two sterile sampling brushes were rotated in the cervix clockwise and counter clockwise for 3 circles respectively. Afterwards, one brush was placed into preserving solution for HPV DNA typing, and the other one was placed into a test tube containing normal saline and centrifuged for 10 min to collect the lower layer which was thereafter transferred into an anticoagulant tube with heparin to detect local lymphocytes using flow cytometry.

High-risk HPV DNA was determined by the second-generation hybridization capture technique7, and the results were analysed by DML2000 system. U. urealyticum and M. hominis were detected using a susceptibility kit for isolation and culture by microbiological assay. *C. trachomatis* was detected by dipstick with latex immunochromatographic assay using a *C. trachomatis* antigen detection kit. Before treatment, 2 ml of fasting cubital venous blood was taken to determine the numbers of T lymphocyte subsets (including CD3+, CD4+ and CD8+ cells) using a human T lymphocyte subset assay kit (XY-1002, Shanghai Xin Yu Biotechnology Co., Ltd., China). Without being frozen, the collected blood was immediately mixed with antibody, incubated at room temperature for 20 min in dark, added red blood cell lysate, incubated at room temperature for 10 min in dark, and centrifuged at 2,000 r/min for 10 min to discard the supernatant. The obtained cells were washed with PBS and resuspended for detection.

### Statistical analysis

All data were analysed by SPSS 16.0 software. The categorical data were expressed as mean ± standard deviation, and intergroup comparisons were performed by the student t test. The numerical data were expressed as rate, and intergroup comparisons were conducted with the chi-square test. P<0.05 was considered statistically significant.

## Results

### Baseline clinical data

The 600 patients were then divided into a high-risk HPV group (high-risk HPV positive) (n=246) and a control group (high-risk HPV negative) (n=354). The high-risk HPV group was aged between 24 and 63 years old, (44.15±7.72) on average. The body mass index (BMI) was (24.02±2.32) kg/m^2^, and 35 patients had diabetes mellitus. The control group was aged from 23 to 66 years old, (44.09±7.52) on average. BMI was (23.87±2.11) kg/m^2^, and 51 patients had diabetes mellitus. There was no significant difference between the baseline clinical data of the two groups (P>0.05).

### HPV infection results

The rate of high-risk HPV infection was 41.0% (246/600), including 140 cases of HPV16 subtype, accounting for 56.9%, followed by HPV52 subtype, accounting for 13.8% (Table 1).

**Table 1:** HPV subtypes

HPV subtype	Percentage n (%)
HPV16	140 (56.9%)
HPV18	18 (7.3%)
HPV31	16 (6.5%)
HPV33	14 (5.7%)
HPV51	8 (3.3%)
HPV52	34 (13.8%)
HPV53	10 (4.1%)
HPV54	6 (2.4%)

### Association between HPV infection and T cell subset

The CD4+ T cell number and CD4+/CD8+ ratio of the high-risk HPV group was significantly lower than those of the control group (P<0.05), but the two groups had similar numbers of CD3+ and CD8+ T cells (Table 2).

**Table 2:** Association between HPV infection and T cell subset

	Case No.	CD3+ T cell (%)	CD4+ T cell %	CD8+ T cell (%)	CD4+/CD8+ ratio
High-risk HPV group	246	65.17±6.19	33.18±2.36	18.46±1.78	1.84±0.32
Control group	354	65.03±6.42	38.96±3.02	18.21±1.54	2.65±0.34
t value		0.267	25.151	1.834	29.397
P value		0.790	<0.0001	0.067	<0.0001

### Relationship between HPV infection and vaginal flora

In the high-risk HPV group, the positive rates of Lactobacillus, C. trachomatis, M. hominis, mycetes, U. urealyticum and bacterial vaginosis were significantly higher than those of the control group (P<0.05). There was no significant difference in the positive rates of trichomonads between the two groups (P>0.05) (Table 3).

**Table 3:** Relationship between HPV infection and vaginal flora

	Case No.	*Lactobacillus*	*Chlamydia*	*Mycoplasma hominis*	*Ureaplasma urealyticum*	Bacterial vaginosis	Mycetes	Trichomonads
High-risk HPV group	246	72 (29.3)	52 (21.1)	70 (28.5)	196 (78.9)	86 (35.0)	46 (18.7)	24 (9.8)
Control group	354	20 (5.6)	18 (5.1)	68 (19.2)	162 (45.8)	46 (13.0)	42 (11.9)	26 (7.3)
Chi-square value		62.366	36.296	7.006	69.359	18.259	5.417	1.105
P value		<0.0001	<0.0001	0.008	<0.0001	<0.0001	0.020	0.293

### Multivariate logistic regression analysis of risk factors for HPV infection

Multivariate logistic regression analysis revealed that C. trachomatis and U. urealyticum were independent risk factors for high-risk HPV infection (P<0.05) (Table 4).

**Table 4:** Multivariate logistic regression analysis of risk factors for HPV infection

Factor	Beta value	Wald value	Odds ratio	95% confidence interval	P value
*Chlamydia*	1.596	4.211	4.968	1.092-10.198	0.039
*Ureaplasma urealyticum*	0.861	11.263	2.422	1.437-3.901	<0.0001
*Lactobacillus*	3.530	3.425	2.843	0.745-12.716	0.071
*Mycoplasma hominis*	1.456	0.628	3.178	0.782-3.155	0.438
Mycetes	1.123	2.498	3.029	0.842-9.856	0.126
CD4+ T cell	1.843	0.317	5.475	0.675-2.467	0.493
CD4+/CD8+ ratio	1.723	0.546	5.230	0.542-2.155	0.528

## Discussion

In this study, the rate of high-risk HPV infection was 41.0% (246/600), including 140 cases of HPV16 subtype, accounting for 56.9%, followed by HPV52 subtype, accounting for 13.8%. The CD4+ T cell number and CD4+/CD8+ ratio of the high-risk HPV group was significantly lower than those of the control group (P<0.05). In the high-risk HPV group, the positive rates of Lactobacillus, C. trachomatis, M. hominis, mycetes, U. urealyticum and bacterial vaginosis were significantly higher than those of the control group (P<0.05). Multivariate logistic regression analysis showed that C. trachomatis and U. urealyticum were independent risk factors for high-risk HPV infection (P<0.05). In clinical practice, therefore, attention should be paid to the control and prevention of *C. trachomatis* and *U. urealyticum* infection, thereby decreasing the high-risk HPV infection rate.

HPV, a DNA virus, is commonly sexually transmitted, which is related to the occurrence of various diseases such as cervical cancer, condyloma acuminata, esophageal cancer, penile cancer and perianal cancer^8^. Currently, there are more than 200 subtypes of HPV, of which more than 40 subtypes are associated with genital tract infections. HPV can be classified into high-risk HPV and low-risk HPV according to the severity of cervical lesions that it causes^9^. High-risk HPV includes subtypes such as HPV16, HPV18, HPV53, HPV52, HPV31, HPV33, HPV51, HPV35, HPV58, HPV66, HPV59, and HPV68; low-risk HPV includes subtypes such as HPV6, HPV42, HPV44, and HPV43. High-risk HPV mainly causes cervical cancer^10^, and low-risk HPV is mainly related to benign lesions such as condyloma acuminata. The high-risk cervical HPV has a relatively high infection rate, and HPV16 is the most common HPV infection subtype of the cervix. The results herein are consistent with previous studies^11^,^12^, indicating that the high-risk cervical HPV has a high infection rate, and the HPV16 subtype is the main infection subtype.

The vaginal micro-ecological flora imbalance increases the expression of HPV, which can cause changes in cervical cytology^13^,^14^. *Lactobacillus* can inhibit the occurrence and development of tumors, activate the mucosal immune system, and enhance the body's immune function^15^. In addition, normal *Lactobacillus* in the vagina has an effect on the prevention of vaginal cancer and cervical cancer. Decreased vaginal lactobacilli can lead to increased opportunistic pathogens, which is conducive to HPV infection and associated with the occurrence of cervical precancerous lesions. After infection, *C. trachomatis* can be adsorbed on the genital tract mucosa, and damage the genital mucosal epithelial cells to cause an inflammatory reaction, and increase local pH of the cervix, which destroys the local immune barrier of the vagina and the cervix, makes for the invasion of pathogenic bacteria, and increases the probability of HPV infection^16^. *U. urealyticum* infection plays an important role in cervical precancerous lesions caused by HPV, which is associated with early cytological changes in the cervix and persistent HPV infection^17^. The infection rate of cervical HPV is also increased when *U. urealyticum* infection occurs. The anaerobic metabolism in bacterial vaginosis can produce nitrosamines that have a carcinogenic effect, and the concentration of phospholipase A2 in cervical secretions of patients with bacterial vaginosis rises^18^, thus increasing the susceptibility of HPV, and promoting the transformation of cervical epithelial cells. Therefore, bacterial vaginosis has a certain relationship with cervical precancerous lesions and cervical cancer^19^,^20^, which was in agreement with the findings of this study.

Both B and T lymphocytes are involved in the immune response in the anti-tumor immune response, of which T lymphocytes play a major role. T lymphocyte-mediated cellular immunity can inhibit and kill tumor cells^21^-^26^. T cells are divided into delayed-type hypersensitivity T cells, killer T cells, cytotoxic T Cells and helper T cells. CD4+ T cells are markers of helper T cells, which play an important role in anti-tumor immunity; CD8+ T cells are divided into cytotoxic T cells and suppressor T cells, whose cytotoxicity can damage vaginal epithelial cells. CD4+/ CD8+ plays an important role in the regulation of immune balance and regulation of immune response. The decrease of CD4+/CD8+ ratio indicates that defective T cell immune function and immune system disorder can cause tumor occurrence^27^,^28^. In this study, multivariate logistic regression analysis found that T lymphocytes were not independent influencing factors of high-risk HPV infection, indicating that cervical immune dysfunction did not directly cause high-risk HPV infection, instead through a synergy with vaginal flora imbalance and other factors^29^.

## Conclusion

In summary, high-risk HPV infection in patients with cervical cancer was associated with vaginal flora and immune function. *C. trachomatis* and *U. urealyticum* are independent risk factors for high-risk HPV infection. Regardless, this study is still limited. The sample size was small. Besides, long-term follow-up was not conducted. Therefore, the results may have bias, and the final treatment outcomes are unclear. Multicentre studies with larger sample sizes and long-term follow-up are in need to validate the findings herein. Particular attention should be paid to the control and prevention of *C. trachomatis* and *U. urealyticum* infection, thereby reducing the high-risk HPV infection rate.
